# Glyoxalase‐1 Overexpression Reverses Defective Proangiogenic Function of Diabetic Adipose‐Derived Stem Cells in Streptozotocin‐Induced Diabetic Mice Model of Critical Limb Ischemia

**DOI:** 10.5966/sctm.2015-0380

**Published:** 2016-08-15

**Authors:** Zhiyou Peng, Xinrui Yang, Jinbao Qin, Kaichuang Ye, Xin Wang, Huihua Shi, Mier Jiang, Xiaobing Liu, Xinwu Lu

**Affiliations:** ^1^ Department of Vascular Surgery, Shanghai Ninth People's Hospital Affiliated to Shanghai JiaoTong University School of Medicine, Shanghai, People's Republic of China; ^2^ Vascular Center of Shanghai JiaoTong University, Shanghai, People's Republic of China

**Keywords:** Glyoxalase‐1, Adipose‐derived stem cells, Angiogenesis, Diabetes, Critical limb ischemia

## Abstract

Adipose‐derived stem cell (ADSC)‐based therapy is promising for critical limb ischemia (CLI) treatment, especially in patients with diabetes. However, the therapeutic effects of diabetic ADSCs (D‐ADSCs) are impaired by the diabetes, possibly through intracellular reactive oxygen species (ROS) accumulation. The objective of the present study was to detect whether overexpression of methylglyoxal‐metabolizing enzyme glyoxalase‐1 (GLO1), which reduces ROS in D‐ADSCs, can restore their proangiogenic function in a streptozotocin‐induced diabetic mice model of CLI. GLO1 overexpression in D‐ADSCs (G‐D‐ADSCs) was achieved using the lentivirus method. G‐D‐ADSCs showed a significant decrease in intracellular ROS accumulation, increase in cell viability, and resistance to apoptosis under high‐glucose conditions compared with D‐ADSCs. G‐D‐ADSCs also performed better in terms of migration, differentiation, and proangiogenic capacity than D‐ADSCs in a high‐glucose environment. Notably, these properties were restored to the same level as that of nondiabetic ADSCs under high‐glucose conditions. G‐D‐ADSC transplantation induced improved reperfusion and an increased limb salvage rate compared D‐ADSCs in a diabetic mice model of CLI. Histological analysis revealed higher microvessel densities and more G‐D‐ADSC‐incorporated microvessels in the G‐D‐ADSC group than in the D‐ADSC group, which was comparable to the nondiabetic ADSC group. Higher expression of vascular endothelial growth factor A and stromal cell‐derived factor‐1α and lower expression of hypoxia‐induced factor‐1α were also detected in the ischemic muscles from the G‐D‐ADSC group than that of the D‐ADSC group. The results of the present study have demonstrated that protection from ROS accumulation by GLO1 overexpression is effective in reversing the impaired biological function of D‐ADSCs in promoting neovascularization of diabetic CLI mice model and warrants the future clinical application of D‐ADSC‐based therapy in diabetic patients. Stem Cells Translational Medicine *2017;6:261–271*

**Significance Statement**

Adipose‐derived stem cells (ADSCs) could be of overarching significance in the management of critical limb ischemia (CLI), especially in CLI patients with diabetes. However, the therapeutic effects of diabetic ADSCs (D‐ADSCs) have been reported to be impaired by hyperglycemia. The present study has proved that genetically modified D‐ADSCs with methylglyoxal‐metabolizing enzyme glyoxalase‐1 (GLO1) could preserve D‐ADSCs and increase cell viability, migration, differentiation, and proangiogenic capacity in high‐glucose conditions. This study has demonstrated that GLO1 overexpression effectively restores the biological function of D‐ADSCs in promoting neovascularization of diabetic CLI mice model and warrants future clinical application of D‐ADSCs‐based therapy in diabetic CLI patients.

## Introduction

Critical limb ischemia (CLI), characterized by ischemic rest pain, nonhealing ulcers, and tissue loss, is the most severe form of peripheral artery disease (PAD) caused by atherosclerosis 1, 2. CLI is a substantial cause of morbidity and health costs and often ends in poor physical function and amputation 3. Type 2 diabetes mellitus (T2DM), which accounts for more than 90% of the total diabetes mellitus (DM) cases, is an important risk factor for the development of all forms of atherosclerosis, increasing both the morbidity and severity of PAD 4 and increasing the amputation rate.

The recent application of stem cell therapy in the clinical management of ischemic diseases has shown encouraging results by increasing neovascularization and blood flow in the ischemic tissue to induce wound perfusion and healing 5 6 7. Because T2DM is often accompanied by obesity, these patients possess a huge reservoir of adipose‐derived stem cells (ADSCs) for stem cell therapy. ADSCs have gained popularity over bone marrow‐derived stem cells (BMSCs) and endothelial progenitor cells for ischemic disease treatment owing to the convenience in obtaining them and the efficiency of their in vitro proliferation without losing stemness. However, T2DM was reported to impair the therapeutic effects of ADSCs through the elevation of intracellular oxidative stress, limiting their proliferation and adhesion and impairing angiogenic potential 8 9 10 11 12. Thus, protecting and reversing dysfunctional diabetic ADSCs (D‐ADSCs) is crucial in the promotion of their clinical application.

Previous studies have demonstrated that the dysfunction of diabetic bone marrow‐derived angiogenic cells is linked to increased dicarbonyl stress. Excessive methylglyoxal (MG) accumulates in cells under hyperglycemic conditions and results in dicarbonyl stress and formation of advanced glycation end products (AGEs) 13. Elevated AGEs further aggravate inflammation and increase oxidative stress by forming reactive oxygen species (ROS) 14, 15. A similar process might contribute to the dysfunction of D‐ADSCs. Glyoxalase I (GLO1) is one of the glyoxalase systems that can detoxify MG and reduce AGE and ROS formation. GLO1 overexpression has been reported to prevent vascular aging and reverse hyperglycemia‐induced angiogenic defects in human endothelial cells, BMSCs, and cardiac stem cells 9, 16, 17. To enhance D‐ADSC‐based stem cell therapy for T2DM patients with CLI, we investigated whether GLO1 overexpression can reactivate dysfunctional D‐ADSCs.

In the present study, we investigated the effects of gene modification by GLO1 lentivirus in the prevention and reversal of hyperglycemia‐induced D‐ADSC dysfunction. We examined whether GLO1 overexpression can effectively protect D‐ADSCs in a high‐glucose environment and restore the angiogenic capacity of D‐ADSCs in a diabetic mice model.

## Materials and Methods

### Animals

Type 2 diabetic mice (BKS.Cg‐m +/+Lepr^db^), wild‐type C57/BL mice, and BALB/c nude mice were purchased from the Shanghai Research Center for Model Organisms (Shanghai, People's Republic of China). All mice were used under a protocol approved by the Animal Experiment and Care Committee of Shanghai Jiaotong University School of Medicine. For all the in vivo experiments, only mice with blood glucose levels greater than 16.7 mmol/l were considered diabetic or hyperglycemic and used for further analysis.

### Isolation, Culture, and Characterization of D‐ADSCs

ADSCs were obtained from the subcutaneous adipose tissues of the inguinal area of diabetic mice (D‐ADSCs) and nondiabetic wild‐type mice (ND‐ADSCs, as a control) and maintained with low glucose (5 mmol/l) Dulbecco's modified Eagle's medium (DMEM), supplemented with 10% fetal bovine serum, 100 U/ml penicillin, and 100 mg/ml streptomycin at 37°C in a 5% CO_2_ incubator, and expanded as previously described 18. To isolate the effect of high‐glucose conditions on D‐ADSCs, DMEM with high glucose (25 mmol/l) were used for culture in all the succeeding experiments. D‐ADSCs and ND‐ADSCs between passage 3 and passage 5 were used for all experiments. To determine the phenotype of ADSCs, the ADSCs in passage 3 were collected, washed with phosphate‐buffered solution (PBS), and incubated with phycoerythrin‐conjugated anti‐mouse antibodies, including CD11b, CD29, CD31, CD44, CD90.1, CD133, and major histocompatibility complex II (MHC‐II) for 25 minutes at 4°C in the dark. Isotype antibodies were used as the control group. After washing the sample three times, the cells were collected for flow cytometry analysis (Beckman Coulter, Fullerton, CA, http://www.beckmancoulter.com).

### Glyoxalase‐1 Overexpression

We used a lentiviral vector for stable gene transfection of D‐ADSCs. A full‐length mouse *Glo1* cDNA was cloned into the pLenti6.3‐MCS shuttle vector (Thermo Fisher Scientific Life Sciences, Waltham, MA, http://www.thermofisher.com), as previously described 19. Lentiviruses were grown by transfecting 293T cells. For their transduction, the D‐ADSCs were incubated with the virus and polybrene for 24 hours. The observation of green fluorescent protein (GFP) expression under fluorescent microscopy and Western blotting were used to assess the transfection efficiency and expression of *GLO1* in ADSCs (G‐D‐ADSCs).

### Cell Viability Assay

The proliferation of D‐ADSCs and G‐D‐ADSCs were compared using cell counting kit‐8 (CCK‐8; Dojindo Laboratories, Kumamoto, Japan, http://www.dojindo.cn) assay. ADSCs were harvested, and 2 × 10^3^ cells were dispensed in a 96‐well plate with high‐glucose DMEM. At 12, 24, 48, and 72 hours, 10 µl of CCK‐8 solution was added to each well in the plate and incubated at 37°C for 2 hours. The optical density of the solution at 450 nm was evaluated using a microplate spectrophotometer (Varioskan; Thermo Fisher). At least four wells were randomly examined each time.

### Measurement of ROS

The intracellular ROS levels of D‐ADSCs, G‐D‐ADSCs, and ND‐ADSCs were determined using the Reactive Oxygen Species Assay Kit (Beyotime Institute of Biotechnology, Haimen, People's Republic of China, http://www.beyotime.com), according to the manufacturer's instructions. In brief, adherent ADSCs cultured in high‐glucose medium were incubated with 6‐carboxy‐2′,7′‐dichlorodihydrofluorescein diacetate at a final concentration of 10 mM for 20 minutes at 37°C and washed three times with HEPES buffer. ROS production in ADSCs was measured fluorometrically with excitation and emission settings at 488 and 525 nm, respectively, and expressed as arbitrary units.

### Measurement of Apoptotic Resistance

To assess the ability of high‐glucose cultured ADSCs to resist cell death induced by an ischemic‐like hypoxic environment, D‐ADSCs, ND‐ADSCs, and G‐D‐ADSCs cultured in 25 mmol/l glucose medium were incubated for 48 hours in a 37°C incubator containing 1% O_2_. After incubation, floating and adherent cells were collected by trypsinization and stained for 15 minutes with Annexin V (Thermo Fisher) and propidium iodide (PI). The percentage of apoptotic cells (Annexin V+/PI−) was quantified by flow cytometry. Moreover, these cells were also collected for Western blot analysis of anti‐apoptotic factor Bcl‐2 and apoptotic factor Bax.

### Multidifferentiation Assays of ADSCs Under High‐Glucose Culture

The D‐ADSCs, ND‐ADSCs, and G‐D‐ADSCs from passage 3 were subjected to stimulation for adipogenic and osteogenic differentiation under high‐glucose conditions according to the supplier's instructions to determine the effect of DM on the ability of stem cells to differentiate into adipocytes and osteocytes, as previously described 18. Osteoblast formation was evaluated after 3 weeks by assessing calcium accumulation using alizarin red (Sigma‐Aldrich, St. Louis, MO, http://www.sigmaaldrich.com). Adipogenic differentiation was assessed using Oil Red O (Sigma‐Aldrich) staining and microscopic observation to visualize the red‐stained oil droplets. Endothelial cell (EC) differentiation was achieved by culturing cells in high‐glucose EGM 2 for 2 weeks 20. The acquisition of the EC phenotype was evaluated by examining the expression of EC specific markers, *Pecam‐1*, *vWF*, and *Cd105*, using reverse transcription quantitative polymerase chain reaction (RT‐qPCR). The primers are listed in supplemental online Table 1.

### Wound Healing Assay and Cell Migration Assay Under High‐Glucose Culture

Scratch wound‐healing assays were performed in six‐well culture plates. Cells at >90% confluence were used; scratches were made using 1‐ml pipette tips, and the wells were washed twice with PBS. The cells were allowed to grow with high‐glucose medium for another 24 hours. Photographs were taken using an Olympus inverted microscope (Olympus Microscopes, Tokyo, Japan, http://www.olympus-ims.com) at 0 and 24 hours. The gap area of the wound was measured using Photoshop software (Adobe Systems Inc., San Jose, CA, http://www.adobe.com), and the data were normalized to the average of the control.

Cell migration assays were also performed using Transwell chambers (24‐well, 8‐μm pore size; Corning, Inc., Corning, NY, http://www.corning.com). High‐glucose DMEM containing 10% serum was used as an attractant in the lower chamber. Next, 1 × 10^5^ cells in DMEM containing 0.5% serum were added to the upper compartment of the insert and allowed to migrate toward the underside of the insert filter at 37°C for 24 hours. The cells that did not migrate through the pores were gently removed with a cotton swab. Cells on the lower side of the insert filter were fixed by 4% paraformaldehyde and stained with 1% crystal violet in 2% ethanol. The cells on the underside of the filter were counted from five randomly selected microscopic views.

### Proangiogenic Analysis of G‐D‐ADSCs Under High‐Glucose Culture

The capacity of ADSCs to stimulate angiogenesis was assessed with tube formation assay using Matrigel (BD Biosciences, San Jose, CA, http://www.bdbiosciences.com), as previously described 21. In brief, conditioned medium was prepared from confluent cultures of D‐ADSCs, ND‐ADSCs, and G‐D‐ADSCs after 48 hours of hypoxic culture (1% O_2_) in high‐glucose medium containing 1% FBS, 100 U/ml penicillin G, 100 µg/ml streptomycin. The concentration of cytokines (vascular endothelial growth factor A [VEGFA], hepatocyte growth factor [HGF], and fibroblast growth factor 2 [FGF2]) was measured using enzyme‐linked immunosorbent assay (R&D Systems, Minneapolis, MN, http://www.rndsystems.com). Furthermore, human umbilical vein endothelial cells were seeded on Matrigel (Corning, Inc.) and incubated in ADSC‐conditioned media for 16 hours at 37°C and 5% CO_2_. Fields were analyzed using an inverted phase contrast microscope. Cumulative tubular growth was determined using ImageJ Pro Plus software (NIH, Bethesda, MD, http://www.www.imagej.nih.gov). To further analyze the proangiogenic capacity, the expression of proangiogenic genes, including *Hif‐1α*, *Vegfa*, and *Sdf‐1α*, were also detected using RT‐qPCR. The primers used are listed in supplemental online Table 1.

### Establishment and Treatment of Diabetic Mouse Hindlimb Ischemia Model

Hyperglycemia was induced in 8‐week‐old male BALB/c mice by intraperitoneal injection of streptozotocin (STZ; 50 mg/kg for 5 days) in 0.05 M sodium citrate. The blood glucose levels were measured 8–10 days after the first STZ injection. Only mice with mean fasting blood glucose greater than 16.7 mmol/l were used. BALB/c mice were then divided into four groups (*n* = 6 for each group) and anesthetized with 3% pentobarbital sodium, 50 mg/kg. The left femoral artery and its branches were ligated through a skin incision in the inguinal region with 9‐0 silk suture. The external iliac artery and all the arteries above it were ligated. The femoral artery was excised from its proximal origin as a branch of the external iliac artery to the distal point at which it bifurcates into the saphenous and popliteal arteries. Laser Doppler imaging analysis was used to validate the limb ischemia model. After arterial ligation and laser Doppler analysis, the ischemic hindlimb was intramuscularly injected with either PBS as the negative control, or D‐ADSCs, G‐D‐ADSCs, and ND‐ADSCs as the positive control (5 × 10^6^ cells; 100 µl; *n* = 6, respectively). The outcome data of limb salvage, limb necrosis (local area), and limb loss (from toe to knee joint) were recorded on day 28.

### Laser Doppler Imaging Analysis

Laser Doppler imaging analysis was performed as described previously 22, 23. In brief, a laser Doppler perfusion imager (moorFLPI; Moor Instruments, Devon, United Kingdom) was used for serial noninvasive physiological evaluation of angiogenesis and neovascularization. The blood flow of the hindlimb was monitored by laser Doppler scanning on days 0, 7, and 28 after treatment. Digital color‐coded images were analyzed to quantify the blood flow in the region from the toe to the knee joint. The perfusion ratios of the ischemic limb to the lateral nonischemic limb in the four groups were analyzed.

### Histological Analysis

Four weeks after cell transplantation and completing the blood flow assessment, the mice were sacrificed and perfusion fixed with 4% paraformaldehyde. The gastrocnemius muscles were then harvested, dehydrated, embedded in paraffin, and sectioned at 5 µm thickness for immunohistochemistry staining with rat anti‐mouse CD31 (Abcam, Cambridge, UK, http://www.abcam.com) to detect the microvessel densities. Cryosections at 10 μm were also cut, fixed in acetone, and immunostained with rat anti‐mouse CD31 and 4′,6‐diamidino‐2‐phenylindole. The samples were photographed with an Olympus microscope equipped with a digital camera (Olympus Microscopes). The microvessels were counted in three areas of highest neovascularization. The average count of three fields was determined in each sample. Coexpression of GFP and CD31 were also analyzed to detect the in vivo endothelial differentiation of D‐ADSCs and G‐D‐ADSCs.

### Statistical Analysis

Data are expressed as the mean ± SEM. Comparisons between groups were performed with one‐way analysis of variance and between individual groups with a two‐tailed Student's *t* test using GraphPad Prism, version 6.0, software (GraphPad, La Jolla, CA, http://www.graphpad.com). Values of *p* < .05 were considered statistically significant.

## Results

### Establishment of GLO1 Overexpression in D‐ADSCs

**Figure 1:**
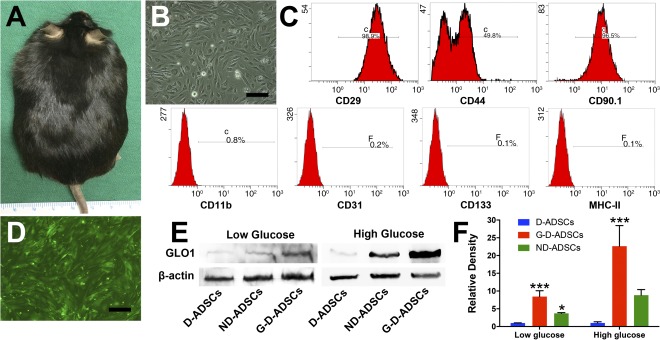
Culture, characterization, and GLO1 transfection of D‐ADSCs. **(A):** Representative image of type 2 diabetes mellitus mouse (BKS.Cg‐m +/+Lepr^db^). **(B):** Representative image of the D‐ADSCs in passage 3. **(C):** Characterization of D‐ADSCs. **(D):** Representative image of GFP+ D‐ADSCs after GLO1 lentivirus transfection. **(E, F):** Western blot showing significantly higher expression of GLO1 expression in G‐D‐ADSCs compared with nontransfected D‐ADSCs and ND‐ADSCs under low‐glucose culture or high‐glucose culture (*n* = 3). ∗, *p* < .05; ∗∗∗, *p* < .001. Scale bar = 100 µm. Abbreviations: D‐ADSCs, diabetic adipose‐derived stem cells; G‐D‐ADSCs, glyoxalase‐1 overexpression in D‐ADSCs; GFP, green fluorescent protein; GLO1, glyoxalase‐1; MHCII, major histocompatibility complex II; ND‐ADSCs, nondiabetic ADSCs.

D‐ADSCs (Fig. 1B) were obtained from BKS.Cg‐m +/+Lepr^db^ mice (Fig. 1A). Flow cytometry analysis showed that D‐ADSCs were strongly positive for the stem cell surface antigens CD29 (98.47% ± 0.54%), CD44 (47.87% ± 1.60%), and CD90.1 (95.00% ± 1.00%), with almost no inflammatory cell, hematopoietic cell, or immune cell (CD11b, CD31, CD133, and MHC‐II) contamination (Fig. 1C). Considering that the impaired function of D‐ADSCs might have been caused by MG accumulation and AGE formation, we performed direct overexpression of GLO1, a detoxifying enzyme of MG, using lentivirus containing GLO1 or GFP as control. After lentiviral transfection, we observed significant expression of GFP in more than 90% of the cells in all groups (Fig. 1D). Western blot showed significantly higher expression of GLO1 in GLO1^+^GFP^+^ D‐ADSCs (G‐D‐ADSCs) compared with either GFP^+^ D‐ADSCs or GFP^+^ ND‐ADSCs, irrespective of whether under low‐glucose conditions or high‐glucose conditions (*p* < .001; Fig. 1E, 1F). These cells were used in all the succeeding experiments.

### GLO1 Overexpression Improves Cell Viability and Reduces ROS Accumulation and Apoptosis of D‐ADSCs Under High‐Glucose Conditions In Vitro

**Figure 2:**
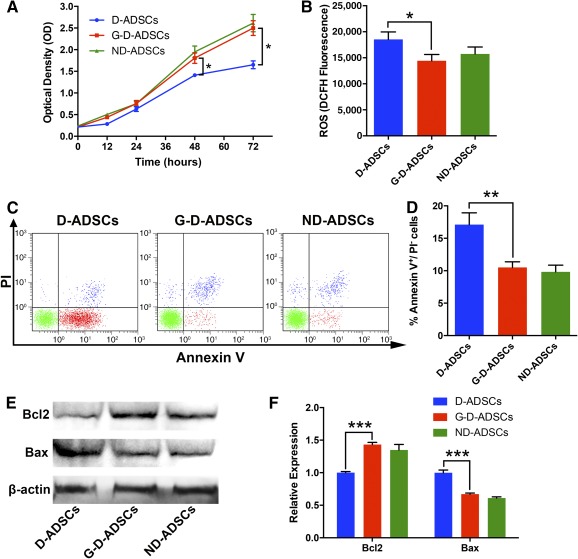
Glyoxalase‐1 overexpression increased the cell viability, decreased the intracellular reactive oxygen species (ROS) accumulation, and reduced the apoptosis rate of D‐ADSC under high glucose conditions. **(A):** Proliferation curve of D‐ADSCs, G‐D‐ADSCs, and ND‐ADSCs. G‐D‐ADSCs showed increased proliferation capacity compared with D‐ADSCs and a capacity comparable to that of ND‐ADSCs under high‐glucose culture (*n* = 4). **(B):** G‐D‐ADSCs showed less intracellular ROS accumulation than D‐ADSCs under high‐glucose conditions (*n* = 3). **(C, D):** Flow cytometry showed fewer apoptotic cells of G‐D‐ADSCs and ND‐ADSCs than D‐ADSCs under high‐glucose and hypoxic conditions (*n* = 3). **(E, F):** Western blot showed higher expression of antiapoptotic factor Bcl2 and lower expression of Bax in G‐D‐ADSCs and ND‐ADSCs than D‐ADSCs under high‐glucose and hypoxic conditions (*n* = 3). ∗, *p* < .05; ∗∗, *p* < .01; ∗∗∗, *p* < .001. Abbreviations: D‐ADSCs, diabetic adipose‐derived stem cells; DCFH, 6‐carboxy‐2′,7′‐dichlorodihydrofluorescein; G‐D‐ADSCs, glyoxalase‐1 overexpression in D‐ADSCs; ND‐ADSCs, nondiabetic ADSCs; PI, propidium iodide.

To investigate the impact of GLO1 overexpression on D‐ADSCs under high‐glucose conditions, we first identified the expansion of these cells using a cell viability assay. D‐ADSCs showed a lagged logarithmic phase and stagnate phase compared with G‐D‐ADSCs and ND‐ADSCs (Fig. 2A), indicating that GLO1 increased the proliferation of D‐ADSCs under high‐glucose conditions. Furthermore, we discovered that the accumulation of ROS in the G‐D‐ADSCs decreased significantly compared with that in the nontransduced D‐ADSCs (0.78‐fold; *p* < .05; Fig. 2B) and was comparable to that in ND‐ADSCs, indicating that GLO1 expression in D‐ADSCs can protect D‐ADSCs from oxidative stress by eliminating ROS.

We then investigated the cell apoptosis of G‐D‐ADSCs and D‐ADSCs under high‐glucose and hypoxic conditions using Annexin V/PI analysis and Western blot. Annexin V/PI analysis showed that G‐D‐ADSCs exhibited a lower rate of apoptosis compared with D‐ADSCs under high‐glucose and hypoxic conditions and was comparable to that of ND‐ADSCs (G‐D‐ADSCs, 12.17% ± 1.17%; D‐ADSCs, 20.10% ± 1.03%; *p* < .01; Fig. 2C, 2D), indicating that GLO1 expression can effectively reduce the apoptosis of D‐ADSCs in high‐glucose and hypoxic environments. We also detected a higher expression of Bcl‐2 and lower expression of Bax in both G‐D‐ADSCs and ND‐ADSCs compared with D‐ADSCs under high‐glucose and hypoxic conditions (*p* < .001), suggesting that GLO1 overexpression can aid in the prevention of apoptosis by enhancing the expression of anti‐apoptotic protein and inhibiting the expression of apoptotic protein. All these results suggest that GLO1 overexpression can improve D‐ADSC survival in high‐glucose and hypoxic conditions.

### GLO1 Overexpression Elevates D‐ADSC Multidifferentiation and Migration Under High‐Glucose Conditions

**Figure 3:**
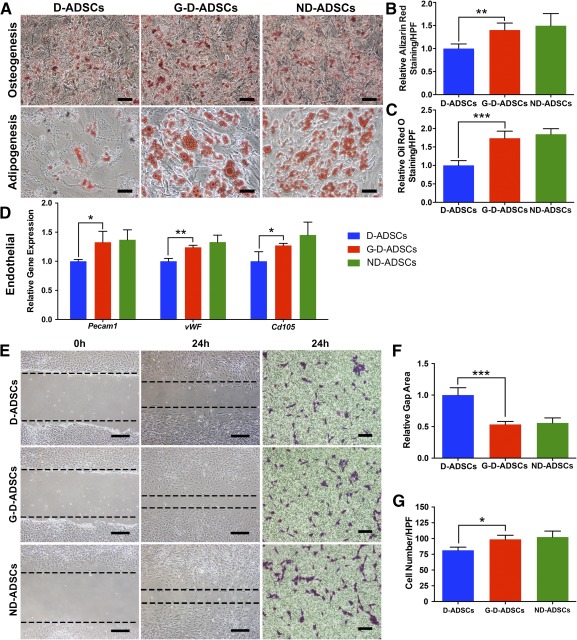
Multidifferentiation and migration analysis of D‐ADSCs and G‐D‐ADSCs. **(A):** Representative images of osteogenesis examined using alizarin red S staining and adipogenesis examined using Oil Red staining after differentiation assays under high‐glucose conditions. **(B):** Significantly more osteogenesis was observed in the G‐D‐ADSC group than in the D‐ADSC group under high‐glucose conditions (*n* = 3). **(C):** Significantly more adipogenesis was observed in the G‐D‐ADSC group than in the D‐ADSC group under high‐glucose conditions (*n* = 3). **(D):** Reverse transcription polymerase chain reaction showed significantly higher expression of specific endothelial gene expression, including *Pecam1*, *vWF*, and *Cd105*, in the G‐D‐ADSC group than in the D‐ADSC group (*n* = 3). **(E):** Representative images of wound healing assay and Transwell assay after 24 hours of culture under high‐glucose conditions. **(F):** Wound healing assay showed that the relative gap area was significantly smaller in the G‐D‐ADSC group than the D‐ADSC group and was comparable to that of ND‐ADSCs under high‐glucose conditions (*n* = 3). **(G):** Transwell assay showed significantly more cells had migrated in the G‐D‐ADSC group than in the D‐ADSC group, which was comparable to that of the ND‐ADSC group under high‐glucose conditions (*n* = 3). ∗, *p* < .05; ∗∗, *p* < .01; ∗∗∗, *p* < .001. Scale bars = 100 µm. Abbreviations: D‐ADSCs, diabetic adipose‐derived stem cells; G‐D‐ADSCs, glyoxalase‐1 overexpression in D‐ADSCs; h, hours; HPF, high‐power field; ND‐ADSCs, nondiabetic ADSCs.

We then detected the multidifferentiation capabilities, including adipogenic, osteogenic, and endothelial cell differentiation of G‐D‐ADSCs under high‐glucose conditions in vitro, using D‐ADSCs and ND‐ADSCs as the control. Osteogenesis in all groups was detected through alizarin red staining and was significantly faster and higher in G‐D‐ADSCs and ND‐ADSCs than in D‐ADSCs (G‐D‐ADSCs to D‐ADSCs, 1.35‐fold; *p* < .01; Fig. 3A, 3B). Similarly, more G‐D‐ADSCs and ND‐ADSCs differentiated into adipocytes at a faster rate than did D‐ADSCs, as proved by Oil Red O staining (G‐D‐ADSCs to D‐ADSCs, 1.74‐fold; *p* < .001; Fig. 3A, 3C). Endothelial cell differentiation was evaluated by comparing the endothelial cell‐specific gene expression among the cell groups using RT‐qPCR. Compared with the D‐ADSC group, the G‐D‐ADSC group and ND‐ADSC group showed higher expression of *Pecam1* (G‐D‐ADSCs to D‐ADSCs, 1.33‐fold; *p* < .05), *vWF* (G‐D‐ADSCs to D‐ADSCs, 1.24‐fold; *p* < .01), and *Cd105* (G‐D‐ADSCs to D‐ADSCs, 1.27‐fold; *p* < .05; Fig. 3D).

As the migration of stem cells from the transplant site to the injured area is essential in stem cell‐based therapy, we detected whether GLO1 overexpression can elevate the migration capacity of D‐ADSCs under high‐glucose conditions. In the scratch assay, G‐D‐ADSCs showed faster healing than D‐ADSCs at 24 hours (relative gap area, 0.50‐fold; *p* < .001; Fig. 3E), comparable to that of ND‐ADSCs. The result of the Transwell assay (Corning, Inc.) showed that more cells migrated through the Transwell membrane in the G‐D‐ADSC group and ND‐ADSC group than in the D‐ADSC group (G‐D‐ADSCs, 98.67 ± 3.76 cells per high‐power field; D‐ADSCs, 77.33 ± 5.36 cells per high‐power field; *p* < .05; Fig. 3F). Overall, these results confirmed that GLO1 overexpression enhanced the migration capacity of D‐ADSCs to the same level as that of ND‐ADSCs in high‐glucose environments.

### GLO1 Overexpression Restores Proangiogenic Activity of D‐ADSCs Under High‐Glucose Conditions In Vitro

**Figure 4:**
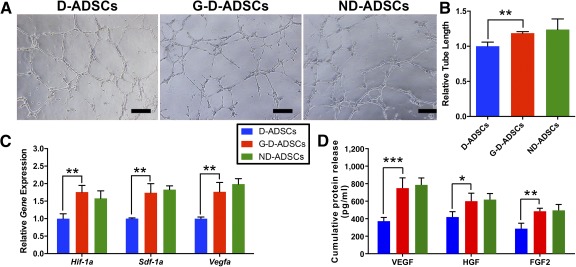
Glyoxalase‐1 overexpression elevates the proangiogenic capacity of D‐ADSCs under high‐glucose conditions. **(A):** Representative images of tube formation of human umbilical vein endothelial cells (HUVECs) seeded onto Matrigel (BD Bioscience) and exposed to high‐glucose conditioned medium prepared from D‐ADSCs, G‐D‐ADSCs, and ND‐ADSCs for 16 hours. **(B):** Cumulative tubular length of HUVECs was higher when cultured in medium collected from G‐D‐ADSCs than from D‐ADSCs and was comparable to that collected from ND‐ADSCs (*n* = 3). **(C):** Expression of proangiogenic genes under hypoxic conditions, including *Hif‐1α*, *Sdf‐1α*, and *Vegfa*, was significantly higher for all in G‐D‐ADSCs than in D‐ADSCs and was comparable to that of ND‐ADSCs (*n* = 3). **(D):** Enzyme‐linked immunosorbent assay showed that proangiogenic factors under hypoxia, including VEGFA, HGF, and FGF2, were all significantly higher in G‐D‐ADSCs than in D‐ADSCs and were comparable to those found in ND‐ADSCs. ∗, *p* < .05; ∗∗, *p* < .01; ∗∗∗, *p* < .001. Scale bars = 100 µm. Abbreviations: D‐ADSCs, diabetic adipose‐derived stem cells; FGF2, fibroblast growth factor 2; G‐D‐ADSCs, glyoxalase‐1 overexpression in D‐ADSCs; HGF, hepatocyte growth factor; ND‐ADSCs, nondiabetic ADSCs; VEGFA, vascular endothelial growth factor A.

To evaluate the impact of direct GLO1 overexpression on the proangiogenic capacity of D‐ADSCs, we performed a tube formation assay using the conditioned medium obtained from D‐ADSCs, G‐D‐ADSCs, and ND‐ADSCs. The conditioned medium from the G‐D‐ADSCs induced higher capillary formation compared with that from D‐ADSCs (1.19‐fold; *p* < .01; Fig. 4A, 4B) and was comparable to that of ND‐ADSCs. G‐D‐ADSCs also showed higher expression of angiogenic genes compared with D‐ADSCs, including *Hif‐1*α (1.76‐fold; *p* < .01), *Sdf‐1*α (1.74‐fold; *p* < .01), and *Vegfa* (1.77‐fold; *p* < .01; Fig. 4C) and was comparable to that of ND‐ADSCs. In addition, we detected higher levels of VEGFA (G‐D‐ADSCs, 749.90 ± 67.50; D‐ADSCs, 372.82 ± 24.26; *p* < .01), HGF (G‐D‐ADSCs, 600.64 ± 52.72; D‐ADSCs, 419.74 ± 34.97; *p* < .01), and FGF2 (G‐D‐ADSCs, 485.59 ± 19.56; D‐ADSCs, 286.44 ± 35.61; *p* < .01) in the conditioned medium of G‐D‐ADSCs and ND‐ADSCs than that of the D‐ADSCs in high‐glucose conditions (Fig. 4 D). These results indicated that direct overexpression of GLO1 can effectively restore the proangiogenic function of D‐ADSCs to the level of ND‐ADSCs in a high‐glucose environment, verifying its importance in the treatment of ischemic patients with T2DM.

### GLO1 Overexpression Protects and Restores the Proangiogenic Activity of D‐ADSCs in Streptozotocin‐Induced Diabetic BALB/c Mice Hindlimb Ischemia Model

**Figure 5:**
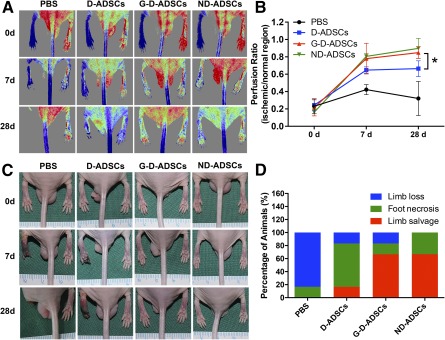
Blood flow reperfusion and outcome of diabetic critical hindlimb ischemia mice model after stem cell therapy. **(A):** Representative images of blood reperfusion measured by laser Doppler analysis at days 0, 7, and 28 after ligation and stem cell therapy (red indicating highest perfusion and blue, lowest perfusion). **(B):** Blood reperfusion data are presented as the average ratio of ischemic to nonischemic limb (from knee to toe) blood flow. G‐D‐ADSC treatment achieved better blood reperfusion than D‐ADSC treatment and was comparable to that with ND‐ADSC treatment (*n* = 6). **(C):** Representative images of ischemic limbs from different groups at days 0, 7, and 28 after ligation and stem cell therapy. **(D):** Outcome data included limb loss, foot necrosis, and limb salvage of mice in all groups (*n* = 6). ∗, *p* < .05. Abbreviations: d, days; D‐ADSCs, diabetic adipose‐derived stem cells; G‐D‐ADSCs, glyoxalase‐1 overexpression in D‐ADSCs; ND‐ADSCs, nondiabetic ADSCs; PBS, phosphate‐buffered saline.

To further validate the restored angiogenic capacity of D‐ADSCs through GLO1 overexpression, we established a diabetic hindlimb ischemia mice model and intramuscularly injected the ischemic hindlimbs with PBS, D‐ADSCs, G‐D‐ADSCs, or ND‐ADSCs (5 × 10^6^ cells, 100 µL; *n* = 6) postoperatively. Blood reperfusion recovered significantly better in the G‐D‐ADSC and ND‐ADSC groups than in the D‐ADSC group (G‐D‐ADSCs, 0.85 ± 0.07; D‐ADSCs, 0.66 ± 0.09; *p* < .05), with nearly no blood reperfusion observed in the PBS group (Fig. 5A, 5B). These findings indicate that GLO1 overexpression can effectively boost the therapeutic blood reperfusion induced by D‐ADSCs transplantation under hyperglycemic conditions in vivo. Consistent with the laser Doppler perfusion imaging results, less limb necrosis or loss and a higher limb salvage rate were observed in the G‐D‐ADSC and ND‐ADSC groups than in the D‐ADSC group. In contrast, nearly all the mice in the PBS group experienced limb loss or necrosis (Fig. 5C, 5D). These results indicate that GLO1 overexpression in D‐ADSCs can significantly improve the prognosis of diabetic hindlimb ischemia.

**Figure 6:**
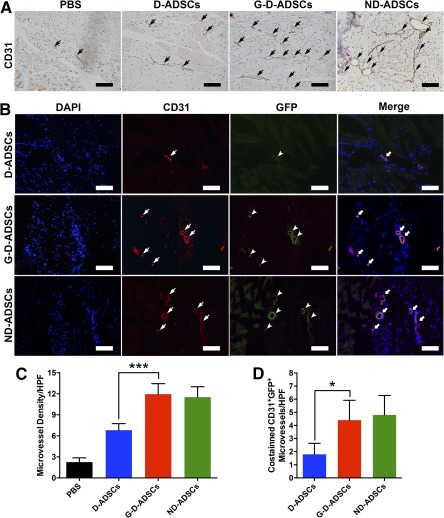
Histological analysis of neovascularization in the ischemic hindlimbs and outcome of transplanted ADSCs. **(A, C):** The microvessel density was significantly higher in the G‐D‐ADSC group and ND‐ADSC group than in the D‐ADSC group (*n* = 3). **(B, D):** Representative images of CD31 (red, indicating microvessel), GFP (green, location of transfected ADSCs), and DAPI (blue, location of nuclei) in D‐ADSC group, G‐D‐ADSC group, and ND‐ADSC group. Significantly more costained CD31^+^GFP^+^ microvessels were observed in the G‐D‐ADSC group and ND‐ADSC group than in the D‐ADSC group (*n* = 3). ∗, *p* < .05; ∗∗∗, *p* < .001. Scale bars = 100 µm. Abbreviations: D‐ADSCs, diabetic adipose‐derived stem cells; DAPI, 4′,6‐diamidino‐2‐phenylindole; G‐D‐ADSCs, glyoxalase‐1 overexpression in D‐ADSCs; GFP, green fluorescent protein; HPF, high‐power field; ND‐ADSCs, nondiabetic ADSCs; PBS, phosphate‐buffered saline.

We further detected the in vivo proangiogenic capacity of direct GLO1 overexpression in D‐ADSCs through histological analysis of microvessel densities (Fig. 6A). The histological results revealed that G‐D‐ADSCs significantly increased the microvessel densities in the ischemic hindlimb compared with that in D‐ADSCs (G‐D‐ADSCs, 10.72 ± 0.36; D‐ADSCs, 6.80 ± 0.42; *p* < .001; Fig. 6C) and was comparable to ND‐ADSCs, showing that GLO1 overexpression can increase the ability of D‐ADSCs to stimulate the growth of microvessels. Furthermore, costaining of CD31 and GFP was performed to detect the in vivo differentiation of ADSCs (Fig. 6B). Significantly more costained microvessels were observed in the G‐D‐ADSC groups than in the D‐ADSC group (G‐D‐ADSCs, 4.40 ± 0.68; D‐ADSCs, 1.80 ± 0.37; *p* < .01; Fig. 6D), which was also comparable to the level in ND‐ADSCs. These results indicate that GLO1 overexpression enhances the differentiation of D‐ADSCs into endothelial cells and participation in the formation of angiogenesis under hyperglycemic conditions in vivo. Therefore, direct GLO1 overexpression can significantly enhance D‐ADSC‐induced angiogenesis in diabetic ischemic tissues in vivo, exhibiting a great possibility in the promotion of stem cell therapy for treating diabetic CLI patients.

### Cytokine Analysis of the Ischemic Tissue

**Figure 7:**
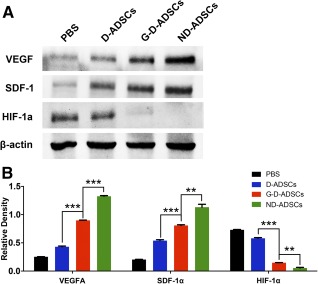
Protein expression of proangiogenic factors in hindlimb tissue. **(A):** Western blot images of relative protein levels of proangiogenic cytokines, including VEGFA, SDF‐1α, and HIF‐1α, in ischemic hindlimb tissue 4 weeks after ligation and stem cell therapy. **(B):** Significantly higher expression of VEGFA and SDF‐1α observed in G‐D‐ADSC group than in the D‐ADSC group but lower expression than in the ND‐ADSC group. The tissue of the G‐D‐ADSC group showed lower expression of HIF‐1α than did tissue in the D‐ADSC group but higher expression than that in the ND‐ADSC group (*n* = 3). ∗∗, *p* < .01; ∗∗∗, *p* < .001. Abbreviations: D‐ADSCs, diabetic adipose‐derived stem cells; G‐D‐ADSCs, glyoxalase‐1 overexpression in D‐ADSCs; HIF‐1α, hypoxia‐induced factor‐1α; ND‐ADSCs, nondiabetic ADSCs; PBS, phosphate‐buffered saline; SDF‐1α, stromal cell‐derived factor‐1α; vascular endothelial growth factor A.

Previous studies have revealed that the angiogenic function of stem cells largely depends on their autocrine and paracrine abilities. Thus, we investigated the proangiogenic function of the G‐D‐ADSCs and D‐ADSCs in vivo by analyzing the proangiogenic factors in the ischemic muscles. Cytokine analysis of the tissues revealed that the VEGFA and stromal cell‐derived factor‐1α (SDF‐1α) content of the hindlimb muscle of the G‐D‐ADSC and ND‐ADSC groups were significantly higher than that of the D‐ADSC group (*p* < .001; Fig. 7A, 7B). However, the paracrine capacity of the G‐D‐ADSCs in vivo was still not at par with that of the ND‐ADSCs. The level of hypoxia‐induced factor‐1α (HIF‐1α), which is upregulated during ischemia and hypoxia, was significantly lower in the G‐D‐ADSC and ND‐ADSC groups than in the D‐ADSC group 4 weeks after ligation (*p* < .001; Fig. 7A, 7B), indicating that G‐D‐ADSCs significantly improved hindlimb hypoxia compared with D‐ADSCs under hyperglycemic conditions. All these results indicate that GLO1 overexpression can significantly improve the angiogenic capacity of D‐ADSCs under DM or hyperglycemic conditions in vivo through paracrine function.

## Discussion

DM or hyperglycemia impairs the proangiogenic function of D‐ADSCs, limiting their application in the treatment of ischemia of PAD accompanied by DM. The present study revealed a molecular target (GLO1) that can protect and restore the biological function of D‐ADSCs, by reducing intracellular ROS accumulation and increasing their resistance to high‐glucose conditions, including their cell viability, differentiation, migration, and proangiogenic capacities in vitro at the same level as that of nondiabetic ADSCs. Moreover, GLO1 overexpression significantly increased the therapeutic angiogenic efficiency of D‐ADSCs in hyperglycemic hosts, showing higher neovascularization and angiogenic factor levels and a better prognosis for the diabetic ischemic model. The present study has provided a solution concerning the restoration of impaired angiogenic capacity of transplanted diabetic ADSCs and promotion of diabetic stem cell‐based therapy for diabetic hosts.

DM is an important risk factor that contributes to the development and severity of all forms of atherosclerosis, including PAD, coronary artery disease, and cerebrovascular disease. DM also increases the risk of ischemic stroke, causes poorer clinical outcomes for PAD patients, and accounts for 60% of nontraumatic lower limb amputations 24, 25. Autologous stem/progenitor cell therapy has recently been developed as a novel option for regenerative medicine for PAD. As it can be obtained using a relatively simple, minimally invasive, and inexpensive procedure, adipose tissue represents an alternative source of multipotent mesenchymal stem cells. T2DM patients account for approximately 90%–95% of total DM patients and have high chances of being obese. Consequently, ADSC is considered a better seed cell for T2DM patients.

Recent studies have revealed that DM and hyperglycemia significantly inhibit BMSC‐mediated hindlimb ischemia repair, cardiac stem cell (CSC)‐mediated cardiac repair, and the neovascularization process. However, contraindications concerning the influence of DM on ADSCs remain. Dentelli et al. previously reported that a diabetic milieu promoted OCT4 and NANOG production, both of which are recognized as crucial transcriptional regulators of stem cell self‐renewal during embryogenesis in human visceral‐derived adipose stem cells, suggesting that high‐glucose preconditioning is a beneficial factor that improves ADSC survival ex vivo 26. However, several other recent studies have suggested that D‐ADSCs exhibit impaired viability and differentiation, including EC and angiogenic differentiation potential 10, 21, 27. The underlying mechanism for DM‐induced ADSC dysfunction remains unclear. It was reported that hyperglycemia can induce the production of ROS in CSCs, leading to increased inflammation and oxidative stress in ischemic tissues and possible activation of apoptosis of CSCs under high‐glucose conditions 28, causing a restrained healing process and more tissue damage. Thus, the elimination of ROS might be a critical factor in the restoration of the effectiveness of stem cell therapy. In the presence of glutathione, MG, which plays a key role in increasing ROS production, forms a hemithioacetal, which is irreversibly converted to lactoylglutathione by GLO1. Lactoylglutathione then forms the substrate for GLO2, which produces D‐lactic acid. This pathway is the principal means of detoxifying excess MG and preventing AGE formation. Therefore, overexpression of GLO1 might possibly restore the capabilities of the impaired D‐ADSCs.

At present, no study has yet focused on the improvement of the function of D‐ADSCs, which is essential in promoting their clinical use for diabetic patients. Our present study provides evidence that GLO1 overexpression can effectively boost cell viability and decrease the apoptosis of D‐ADSCs. The upregulated Bcl‐2/downregulated Bax and a lower apoptotic index of D‐ADSCs under high‐glucose and hypoxic conditions after GLO1 overexpression indicate that GLO1 can possibly protect stem cells by resisting apoptosis under apoptotic conditions. G‐D‐ADSCs also increased the tube formation and expression of previously downregulated proangiogenic genes, including *Hif‐1α*, *Sdf‐1α*, and *Vegfa*, all of which are related to angiogenesis under hypoxia. As *Hif‐1α* expression is impaired by high glucose in diabetes 29, the increased angiogenic capacity of G‐D‐ADSCs might be related to the elimination of ROS and decreased oxidative stress caused by GLO1 that can lead to upregulation of *Hif‐1α* and, consequently, elevated expression of *Sdf‐1α* and *Vegfa*. This observation might be the underlying mechanism of GLO1 overexpression‐mediated restoration of the proangiogenic capacity of D‐ADSCs. Furthermore, G‐D‐ADSCs injected in the ischemic hindlimb muscles of the hyperglycemic host showed higher microvessel densities compared with D‐ADSCs, suggesting that genetic modification of D‐ADSCs with GLO1 is an effective approach in preserving D‐ADSC‐mediated neovascularization in CLI cases. Significantly more costained GFP+/CD31+ vessels were observed in the G‐D‐ADSC group, indicating that GLO1 overexpression also preserved endothelial differentiation of D‐ADSCs under hyperglycemic conditions in vivo. This phenomenon was accompanied by higher expression of VEGFA and SDF‐1α in the ischemic tissues of the G‐D‐ADSC group. However, we found that the in vivo paracrine capacity of the G‐D‐ADSCs was still not at the same level as that of the ND‐ADSCs. This might have resulted from the possible blood glucose fluctuation and other diabetes‐related factors in the diabetic mice model and that the D‐ADSCs were impaired before they were obtained and expanded. Further studies are needed to clarify this. Nevertheless, the decreased expression of Hif‐1α during the fourth week validated that less hypoxia was present in the G‐D‐ADSC transplanted muscles compared with the D‐ADSC group. Taken together, the D‐ADSCs with GLO1 overexpression showed effects that approximate that of ND‐ADSCs, indicating that GLO1 overexpression could reverse the dysfunction of D‐ADSCs under high‐glucose conditions. All these results indicate that GLO1 overexpression improves the paracrine function of angiogenic factors of D‐ADSCs under hyperglycemic conditions in vivo and is effective in improving compromised D‐ADSCs for diabetic CLI treatment.

However, the present study only focused on reversing the dysfunctional D‐ADSCs under hyperglycemia and used an STZ‐induced T1DM mice model. Further study of the effects of other pathophysiological factors related to T1DM or T2DM on D‐ADSCs is still needed. The specific underlying mechanisms of GLO1 overexpression in the promotion of angiogenic function of diabetic somatic stem cells should also be thoroughly defined in future research. Also, most parts of the present study were performed under high‐glucose conditions, and we only focused on protecting the D‐ADSCs under hyperglycemic condition. Further study of the effectiveness of GLO1 overexpression on D‐ADSCs and how these cells can be repaired at the same level as that of ND‐ADSCs under normoglycemic condition will further promote the clinical feasibility of D‐ADSC transplantation. Also, the present study examined only short‐term results and concentrated on increasing the therapeutic neovascularization efficiency of diabetic ADSC‐based therapy. High GLO1 expression was also reported to be closely related to a series of tumors, such as hepatocellular carcinoma 30, 31, gastric cancer 32, cutaneous neoplasms 33, and prostate cancer 34. Long‐term studies are still required to determine the safeness of GLO1 overexpression in ADSCs. In our study, no neoplasm formation was detected, and no mice died during our experiments. Notwithstanding its limitations, the present results suggest that modifying diabetic ADSCs with GLO1 can effectively elevate the therapeutic efficiency and promote stem cell therapy for the treatment of diabetic patients with ischemic diseases.

## Conclusion

Our study has provided direct evidence that direct GLO1 overexpression can effectively restore the impaired cell viability, migration, differentiation, and proangiogenic capacity of D‐ADSCs both in vitro and in vivo, leading to better prognosis of diabetic ischemic models. Thus, direct GLO1 overexpression can be used as an essential therapeutic target in stem cell‐based therapy for diabetic CLI patients.

## Author Contributions

Z.P.: conception and design, collection and assembly of data, data analysis and interpretation, manuscript writing; X.Y.: collection and assembly of data, manuscript writing; J.Q. and K.Y.: administrative support, data analysis and interpretation; X.W. and H.S.: provision of study material or patients, administrative support; M.J.: data analysis and interpretation; X. Liu and X. Lu: conception and design, data analysis and interpretation, manuscript writing, final approval of manuscript.

## Disclosure of Potential Conflicts of Interest

The authors indicated no potential conflicts of interest.

## Supporting information

Supporting Information
