# Supplementary material for: Glyoxalase‐1 Overexpression Reverses Defective Proangiogenic Function of Diabetic Adipose‐Derived Stem Cells in Streptozotocin‐Induced Diabetic Mice Model of Critical Limb Ischemia
Source: Stem Cells Transl Med. 2016 Aug 15;6(1):261–71. doi: 10.5966/sctm.2015-0380 (PMC5442730; doi:10.5966/sctm.2015-0380)
Supplement: Supplementary file 1 — Supporting Information [file SCT3-6-261-s001.pdf]

Supporting Information Table 1 Primers sets used in the present study

Target	Forward	Reverse
Glo-1	ATGGCAGAACCGCAGCCC	CATTAAGGTTGCCATTTTGTTAGGA
Pecam1	CAAACAGAAACCCGTGGAGATG	ACCGTAATGGCTGTTGGCTTC
vWF	GGCTGTGCGGTGATTTTAACAT	CGTTTACACCGCTGTTCCTCA
Cd105	CTCCATGCGCCTGAACATC	GTGATACCCAGTACAGAGGGCAG
Vegfa	CATCTTCAAGCCGTCCTGTGT	CTCCAGGGCTTCATCGTTACA
Sdf-1α	TACAGATGCCCATGCCGA	CTGAAGGGCACAGTTTGGAG
Hif-1α	GTCTCGAGATGCAGCCAGAT	TCACCAGCATCCA GAAGTTTC
β-actin	TGTTACCAACTGGGACGACA	CTGGGTCATCTTTTCACGGT
